# Samuel Goldflam (1852–1932)

**DOI:** 10.1007/s00415-012-6471-0

**Published:** 2012-03-29

**Authors:** Andrzej Grzybowski, Jarosław Sak

**Affiliations:** 1 Department of Ophthalmology, Poznań City Hospital, ul. Szwajcarska 3, 61-285 Poznań, Poland; 2 Medical Faculty, University of Warmia and Mazury, Olsztyn, Poland; 3 Department of Ethics and Human Philosophy, Medical University of Lublin, Szkolna 18, 20-124 Lublin, Poland

**Fig. 1:**
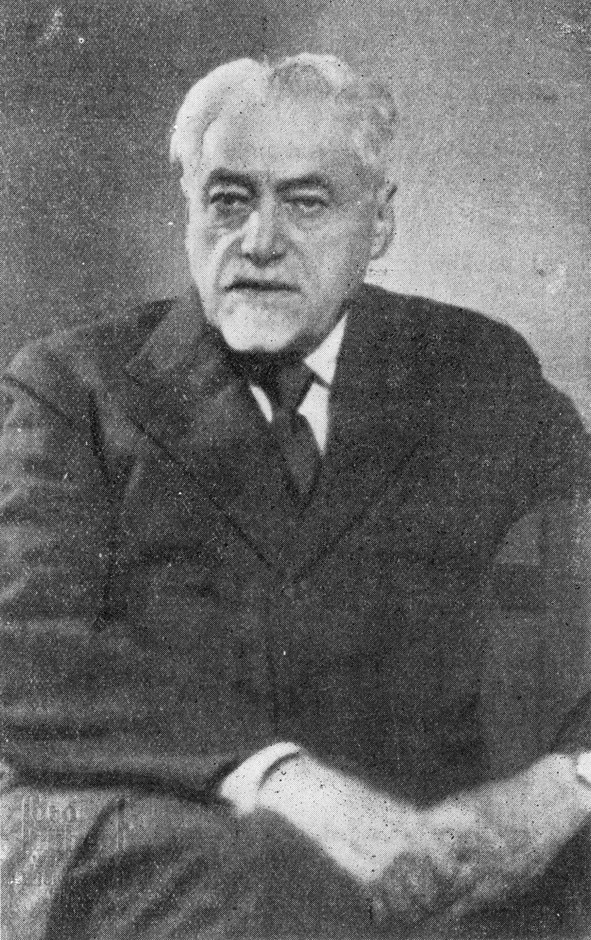
Samuel Goldflam (1852–1932). Reprinted from: Warszawskie Czasopismo Lekarskie 1932, 9 (40): title page

The year 2012 marks the 80th anniversary of the death of Samuel Goldflam. One of his achievements is the description of myasthenia gravis, a disease which has also become known as Erb-Goldflam disease [2, 9, 10] (Fig. 1).

Samuel Goldflam was born in Warsaw on February 15, 1852; his father was the Jewish merchant Wolf Goldflam. After graduating from secondary school (gymnasium), he studied between 1870 and 1875 at the Medical Faculty of the Imperial University of Warsaw [1, 8]. In 1875, he received his medical degree and started work at the Holy Spirit Hospital in the clinic for internal diseases of Prof. Vilém Dušan Lambl (1824–1895). From 1876, he worked as an assistant at the Therapeutic Clinic for internal diseases of the Imperial University of Warsaw [1, 8]. In 1878, Goldflam became head of the department in Lambl’s clinic. During this time, he also accepted patients free of charge in his outpatient practice on Długa Street in Warsaw. In 1882, he went abroad to perfect his medical knowledge under the tutelage of Carl Friedrich Otto Westphal (1833–1890) in Berlin and of Jean-Martin Charcot (1825–1893) in Paris. After his return to Warsaw in 1883, he opened an internal and neurological clinic for the indigent in his apartment located at 10 Graniczna Street, which he ran for almost four decades until 1922 [8]. During this time, he collected a large amount of knowledge from clinical observations. After the closure of the polyclinic, Samuel Goldflam worked as a volunteer in the neurological department of the Old Order (Jewish) Hospital in the Czyste district in Warsaw [8]. He was one of the founders of the Warsaw Neurological Society and its first president between 1921 and 1923. Goldflam did not found a family, dedicating his entire life to professional work and social activities. He cooperated in social support of poor people with the leading pre-war Jewish educator and physician Janusz Korczak, who was also known as Henryk Goldschmidt (1878–1942). Together these two contributed to the reactivation of the Berson and Bauman Families’ Children’s Hospital in Warsaw in 1930. In 1908, Goldflam established a hospital for mentally ill patients of Jewish nationality called “Zofiówka” in Otwock near Warsaw [8], of which he was the director until 1926. He was active in several other organizations that provided social support for people of Jewish origin such as the Society for Spreading Education and Culture Among the Jewish Population “Daath”, the Association of Health Protection of the Jewish Population, the Jewish Support Association “Bajs-Lechem”, and the Jewish Society for the Advancement of Fine Arts. He was also a co-founder of the Hebrew University’s Friends Association. In 1925, he was a delegate of the Jewish community of Warsaw to take part in the inauguration ceremony of the Hebrew University in Jerusalem. Goldflam was known for his passion for classical music, especially the works of Wagner and Beethoven. He was a frequent visitor to the Warsaw Philharmonic [8]. He helped in developing the careers of many artists, including Arthur Rubinstein (1887–1982). Samuel Goldflam died on August 26, 1932 at the age of 80 years from a tumor in the mediastinum. He was buried at the Jewish Cemetery, which still exists in Warsaw on Okopowa Street. His collection of art (about 200 pieces) and books was bequeathed to the Hebrew University in Jerusalem.

Throughout his professional life, Samuel Goldflam strived to solve mysteries of neurological and general medical diagnoses with great commitment. He described inequality of knee jerks in tabes dorsalis, pointed out the decrease or disappearance of the Achilles tendon reflex in tabes dorsalis as well as in sciatica, and compared tendon jerks in patients and healthy people both during sleep and wakefulness. Goldflam also attached great importance to studying skin reflexes and noted the antagonistic behavior of skin and tendon reflexes in advanced diabetes (tendon jerks often disappear, while skin reflexes remain strong). In 1930, Goldflam described the pathway of the reflex arc for Rossolimo’s sign: he postulated a reflex center in the spinal cord in the L5–S1 segment and an inhibitory center in the prefrontal and the right middle frontal cortex [7]. Even before experiments conducted a few years later by John Farquhar Fulton (1899–1960), he proved that the descending motor pathways in question do not follow the pyramidal tract, but lie within the vicinity of the lateral funiculus of the spinal cord as a non-pyramidal tract from the premotor area. He also studied the pathways of the pupillary light reflex [5]. In 1893, Goldflam presented three extensive case reports of the disease that would later become known as myasthenia gravis, with a full review of previous descriptions [3, 9]. Among the symptoms of the disease he emphasized muscular fatigue (apokamnosis) as a pathological symptom of this disease. Henry Viets described Goldflam’s work of 1893 [3] as “in many ways the most important paper written in the history of the disease” [10]. In other areas of medicine, Goldflam described the “kidney punch”, known today as the costovertebral angle tenderness (in Polish terminology Goldflam’s symptom) [4]. He also presented the first detailed clinical description and explanation of intermittent claudication, including the paleness of the foot that occurs after active movement as a symptom of this disease (Goldflam–Oehler sign) and the causal role of habitual smoking. Directly after the World War I, he dealt with an epidemic of von Economo encephalitis, which began in Poland in the winter of 1919. Observation of patients with this disease entity inspired Goldflam’s research on the functioning of muscle agonists and antagonists. He described complications after encephalitis, e.g., in parkinsonism [6]. As the first scientist in Poland, he conducted his own microscopic research on the nervous system.

Goldflam was one of the pioneers who shaped contemporary neurology. This especially applies to his observations of myasthenia gravis—a name that would be proposed by Jolly in 1895, 2 years after Goldflam’s seminal description [9, 10]. It is also fitting to underline the sensitivity to the problems of poor patients that guided Samuel Goldflam.
